# Anti-Wrinkling and Anti-Melanogenic Effect of *Pradosia mutisii* Methanol Extract

**DOI:** 10.3390/ijms20051043

**Published:** 2019-02-27

**Authors:** Laura Rojas Lorz, Byong Chul Yoo, Mi-Yeon Kim, Jae Youl Cho

**Affiliations:** 1 Department of Integrative Biotechnology, Sungkyunkwan University, Suwon 16419, Korea; laurisrl@gmail.com; 2 Biomarker Branch, Research Institute, National Cancer Center, Goyang 10408, Korea; yoo_akh@ncc.re.kr; 3 Department of Cancer Biomedical Science, Graduate School of Cancer Science and Policy, National Cancer Center, Goyang 10408, Korea; 4 School of Systems Biomedical Science, Soongsil University, Seoul 06978, Korea

**Keywords:** antioxidant activity, anti-melanogenic effect, anti-wrinkling effect, moisturizing

## Abstract

Ultraviolet (UV) exposure causes skin photoaging leading to skin wrinkling and sagging via production of reactive oxygen species (ROS). For this reason, protection from photoaging is an important feature in cosmeceutical and dermatological products. Natural product-derived biomaterials are highly desired as future possible ingredients, because these biomaterials are often safe and effective. In this study, we aimed to characterize the skin protective activity of *Pradosia mutisii*, traditionally used to treat sunburn and erythema. We determined the free radical scavenging, anti-melanogenic, and moisturizing effects of a methanol extract of *Pradosia mutisii* (Pm-ME) in keratinocytes (HaCaT cells), melanocytes (B16F10 cells), and fibroblasts (human dermal fibroblasts (HDFs)) at non-cytotoxic concentrations. *Pradosia mutisii* methanol extract contains coumaric acid as a major component, and the extract exhibited protective activity against UVB- and H_2_O_2_-induced cytotoxicity. This extract also suppressed the expression of *metalloproteinases* (*MMP*s) and *cyclooxygenase (COX)-2* in HaCaT cells. A reduction of *Sirt-1* expression under UVB- and H_2_O_2_-treated conditions was recovered in HaCaT cells by Pm-ME. This extract displayed significant free radical scavenging activity according to the 2,2′-azino-bis (3-ethylbenzothiazoline-6-sulphonic acid) diammonium salt (ABTS) assay. The Pm-ME also upregulated the expression levels of *hyaluronic acid synthase* (*HAS*) and *transglutaminase-1* (*TGM-1*) in HaCaT cells, indicating a putative moisturizing activity. Interestingly, the expression of collagen type 1 (*Col1A1*) gene and its promoter activity, as assessed by a reporter gene assay, were found to be increased in HDF and HEK293 cells. Similarly, Pm-ME helped recover collagen levels after UVB and H_2_O_2_ treatment in HDFs as well as decreased the synthesis and secretion of melanin from B16F10 melanoma cells, which may indicate a beneficial whitening cosmetic value. The p38 inhibitor SB203580 and the JNK inhibitor SP600125 suppressed *MMP-9* and *COX-2* expression in H_2_O_2_-treated HaCaT cells. Similarly, the ERK inhibitor U0126 inhibited *HAS-2* in Pm-ME/H_2_O_2_-treated HaCaT cells. These findings suggested that inhibition of JNK and p38 and activation of ERK could be targeted by Pm-ME. Therefore, Pm-ME may exert anti-photoaging and anti-melanogenic properties via the regulation of mitogen-activated protein kinase, which could be beneficial in the cosmeceutical industry.

## 1. Introduction

Skin is the largest organ in the body, which serves as a barrier between the organism and the environment, and is responsible for maintaining skin homeostasis and ultimately determining the organism’s survival [1]. Skin is organized with epidermis, dermis with adnexal structures, and subcutaneous fat [2]. In addition, skin is important for continuous communication with immune, neural, and endocrine systems [3], it mostly confers protection against pathogens, chemicals, physical injuries, and ultraviolet (UV) irradiation [4]. Ultraviolet spectra is divided into three zones, UVC (200 to 280 nm), UVB (280 to 320 nm), and UVA (320 to 400 nm) [5]. Of these, UVB is predominantly absorbed by the upper layers of skin epidermis as well as papillary dermis, while UVA penetrates to the reticular dermis with 1000 times lower efficiency at inducing various biological effects, compared to UVB [5]. Although UVC is very reactive and absorbed by the stratum corneum, it is mostly removed by the ozone layer and atmosphere. As a major UV irradiation source, UVB can lead to the production of reactive oxygen species (ROS) such as hydrogen peroxide (H_2_O_2_) via activation of ROS-generating enzymes including NADPH oxidase, xanthine oxidase, and D-amino acid oxidase [6,7,8] and the induction of skin photoaging, which leads to skin wrinkling and sagging [9,10]. In addition, oxidative stress response has been reported to induce various cellular component damage such as cellular membrane lipids, proteins, and nucleic acids [11]. These damaged skin cells are able to initiate inflammatory responses leading to eventual damage manifested in chronically exposed skin [12].

Oxidative stress induces the activation of inflammatory and redox sensitive transcription factors, nuclear factor (NF)-κB and activator protein (AP)-1, and their upstream signaling enzymes including mitogen-activated protein kinases (MAPKs) such as extracellular signal-regulated kinase (ERK), p38, and c-Jun-N-terminal kinase (JNK) in the AP-1 pathway, or as an inhibitor of κBα (IκBα), IκBα kinase (IKKα/β), and AKT in the NF-κB pathway linked to the induction of inflammation and wrinkle formation [13]. Extracellular signal-regulated kinase normally mediates cellular responses related to growth factors, JNK and p38 mediated cellular responses related to cytokines and physical stress [14]. Mitogen-activated protein kinases can also induce the production of proteolytic matrix metalloproteinases (MMPs) [15], which induce collagen degradation thus decreasing skin elasticity [16]. Regarding these components, anti-oxidative components or extracts as ginsenoside, curcumin, epicatechin, asiaticoside, ziyuglycoside I, magnolol, gallic acid, hydroxychavicol, hydroxycinnamic acids, glycyrrhizic acid, mangiferin, mirkoin, rosmarinic acid, tectorigenin, tyrosol, BIOGF1K, and hydroalcoholic extract of *Spartium junceum* L. flowers have been used for the development of anti-skin aging products [17,18,19,20,21]. Because oxidative stress is known as a major cause of human disease and the aging process which affects longevity, secondary bioactive metabolites in human diets with antioxidative properties are considered valuable ingredients [22,23].

Other photoaging-related genes include *cyclooxygenase-2* (*COX-2*) and *Sirt-1*. *Cyclooxygenase-2* is usually overexpressed in premalignant UV-induced skin lesions [24], and its inhibition can decrease malignant transformation in the epidermis [25]. *Sirt-1* expression can prevent cell apoptosis and increase cell survival [26]. Another mechanism of protection against UVB radiation is the production of melanin, a pigment synthesized in melanocytes and further secreted to the keratinocytes in the epidermis layer [1]. Melanin is produced by the oxidation of L-tyrosine and its following conversion to L-dihydroxyphenilalanine (L-DOPA) [6] by catalyzing with the copper-dependent enzyme tyrosinase [27]. Despite its protective function, the excessive production of melanin can generate age spots [28], melisma, and hyperpigmentation [29]. Because of the current trend which considers light complexions as the beauty standard, skin whitening preparations that achieve either hyperpigmented lesions bleaching or skin whitening have become highly desirable in the pharmaceutical and cosmeceutical industries [29]. Thus, considerable effort has been directed towards the development of preparations that reduce melanin synthesis [30]. Because skin aging is associated with loss of skin moisture, an important consideration for maintaining healthy skin is adequate hydration [31]. Hyaluronic acid (HA), a high molecular weight glycosaminoglycan with hydrophilic properties, contributes to the hydration and plastic properties of the skin by regulating the expression of *hyaluronic acid synthases* (*HAS*s) [32]. Another important gene in skin is collagen, which provides support for epidermal structures [33], therefore being responsible for the strength and resilience of skin [34] and whose degradation leads to both skin sagging and wrinkling. In addition, *transglutaminase-1* (*TGM-1*) is an epidermal constitutively expressed enzyme that catalyzes cornified epidermal cell envelope formation, helping to prevent water loss [35,36].

The Sapotaceae family is distributed mainly in the tropical and subtropical regions of Asia and Mesoamerica. Many Sapotaceae family species produce edible fruits of high economic value. These fruits have been used for medicinal purposes. In particular, the seeds are rich in nutrients, vegetable fats, proteins, and other beneficial compounds. For example, mamey oil has traditionally been used in skin care, to treat sunburns and erythema [37]. *Pradosia mutisii* is a member of the Sapotaceae family whose oil has traditionally been used to cure skin scars [38]. However, its properties have yet to be scientifically proven. The aim of this research was therefore to determine the potential value of *P. mutisii* in skin care, cosmetology, and pharmacology.

## 2. Results

### 2.1. Pm-ME Characterization and its Effect on Cell Viability

**Figure 1:**
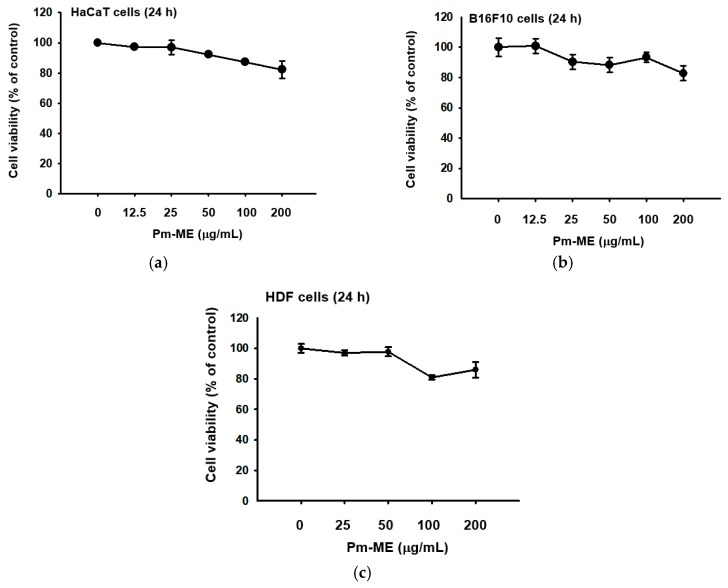

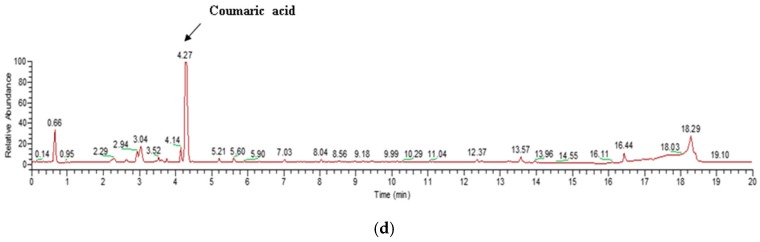
The effect of Pm-ME on the viability of HaCaT, B16F10, and HDF cells, with Pm-ME UPLC profiling. (**a**–**c**) The viabilities of Pm-ME (12.5 to 200 µg/mL)–treated HaCaT, B16F10, and HDF cells were measured via MTT assay. (**d**) The chemical profile of Pm-ME was analyzed using UHPLC and mass spectrometry. The major compound at 4.27 min was revealed to be coumaric acid.

Pm-ME did not block viability up to 100 µg/mL, but slightly decreased viability at 200 µg/mL in HaCaT, B16F10, and human dermal fibroblast (HDF) cells, according to the 3-(4,5-dimethylthiazole-2-yl)-2,5-diphenyltetrazolium bromide (MTT) assay (Figure 1a–c). Using ultrahigh-pressure liquid chromatography (UHPLC) and liquid chromatography (LC)/mass spectrometry, a major compound in Pm-ME was found to be coumaric acid at 4.27 min (Figure 1d).

### 2.2. Protective Effect of Pm-ME against UVB and H_2_O_2_ damage

**Figure 2:**
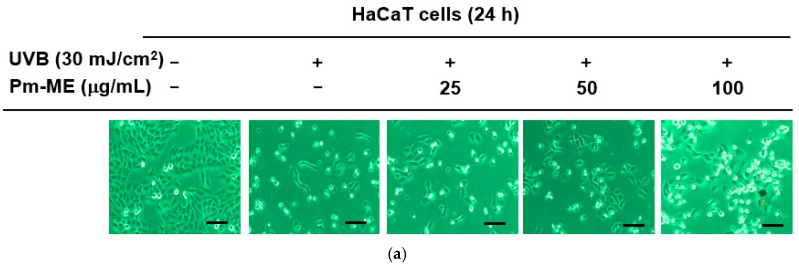

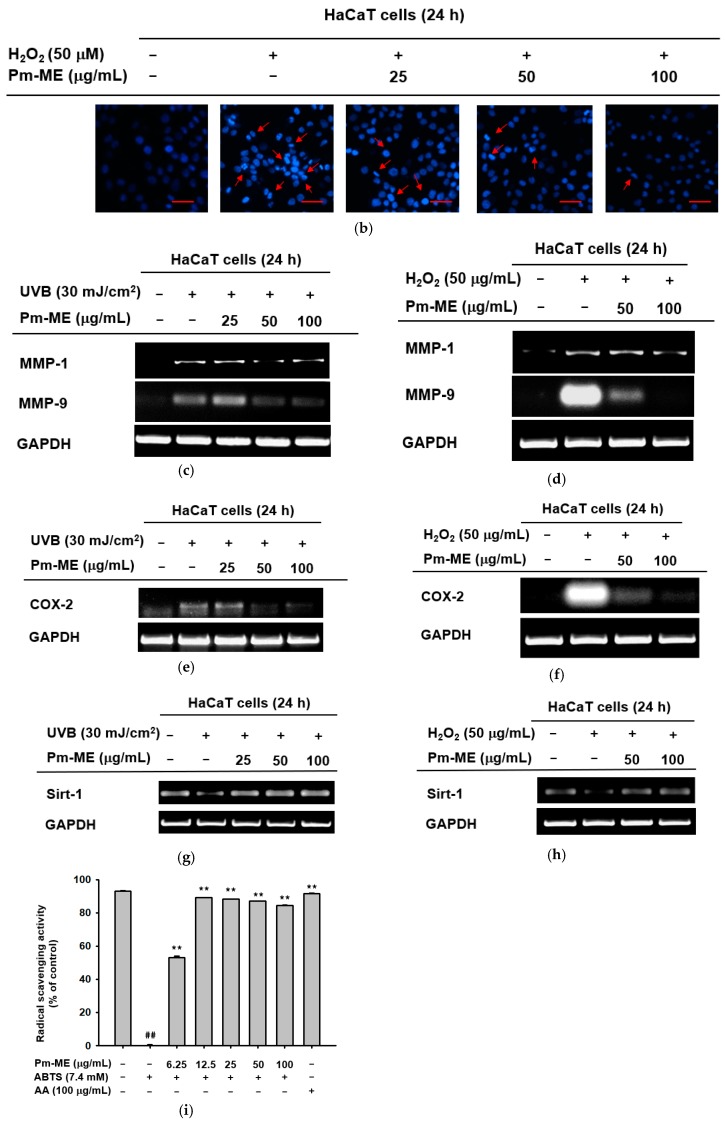
The effect of Pm-ME on UVB- and H_2_O_2_-induced cellular damage, and molecular responses in HaCaT cells. (**a**) HaCaT cells pre-treated with Pm-ME (0–100 µg/mL) for 30 min were subjected to UVB irradiation (30 mJ/cm^2^) and then further incubated for 24 h. Morphological changes, adherence, and numbers of cells were assessed by taking photographs with a digital camera. Scale equivalent to 1 mm (**b**) HaCaT cells pre-treated with Pm-ME (0–100 µg/mL) for 30 min were treated with H_2_O_2_ (50 µM) for 24 h. The generation of apoptotic bodies was examined by 4′,6-diamidino-2-phenylindole (DAPI) staining (Sigma–Aldrich) of the nucleus followed by confocal microscopy. Red arrows indicate apoptotic bodies. Scale equivalent to 100 px (**c**–**h**) Reverse transcription-polymerization chain reaction (RT-PCR) was used to determine the mRNA expression levels of *MMP-1*, *MMP-9*, *COX-2*, and *Sirt-1* from UVB- or H_2_O_2_-treated HaCaT cells pre-treated with Pm-ME. (**i**) The radical scavenging effect of Pm-ME was measured by ABTS assay ** *p* < 0.01 versus an induced group (##). AA: ascorbic acid.

To determine whether Pm-ME protected HaCaT cells against damage caused by UVB- and H_2_O_2_-induced photoaging, cells were pretreated with different concentrations of Pm-ME (0–100 µg/mL) prior to exposure to UVB or H_2_O_2_. Figure 2a shows that UVB irradiation decreased the numbers of adherent HaCaT cells, while Pm-ME increased cell adherence. In addition, cellular damage triggered by H_2_O_2_ was recovered by Pm-ME at 100 µg/mL (Figure 2b). To elucidate whether Pm-ME attenuated UVB- and H_2_O_2_-mediated wrinkle formation, the expression levels of *MMP-1* and *MMP-9* were measured following UVB or H_2_O_2_ treatment in the presence or absence of Pm-ME. Pm-ME inhibited the expression of *MMP-1* and *MMP-9* under both conditions (Figure 2c,d). We next examined whether Pm-ME suppressed free radical-induced inflammatory responses by measuring mRNA levels of *COX-2*. Under UVB- or H_2_O_2_-treated conditions, mRNA levels of *COX-2* were strongly suppressed by Pm-ME at 100 µg/mL (Figure 2e,f). We then measured mRNA levels of the anti-aging gene, *Sirt-1*, to determine its impact on photoaging. Both UVB and H_2_O_2_ reduced Sirt-1 expression, whereas Pm-ME remarkably restored its expression at 50 and 100 µg/mL (Figure 2g,h). Because Pm-ME suppressed certain molecular and cellular responses in HaCaT cells treated with UVB or H_2_O_2_, we next examined whether this extract had direct anti-oxidative activity using 2,2′-azino-bis (3-ethylbenzothiazoline-6-sulfonic acid) ABTS assay, in which Pm-ME showed dose-dependent free radical scavenging activity (Figure 2i).

### 2.3. Moisturizing and Collagen-Increasing Effects of Pm-ME 

**Figure 3:**
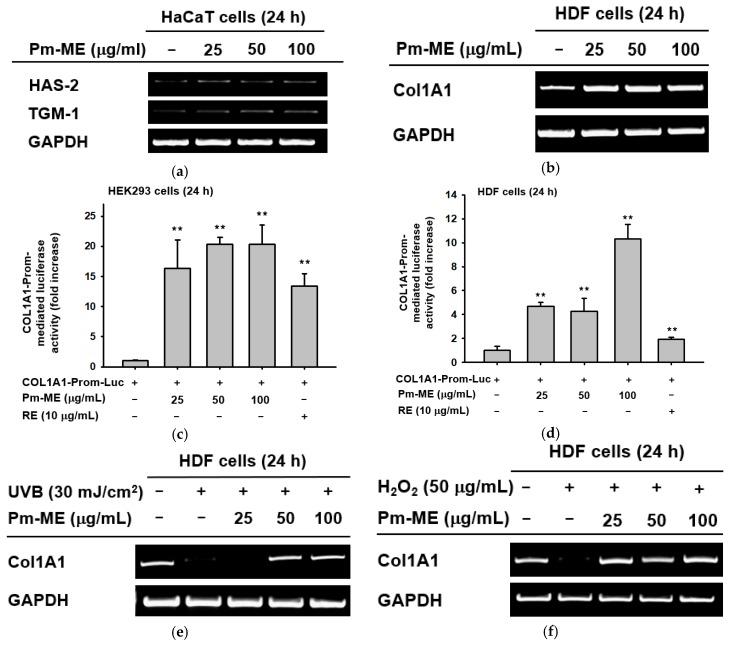
Moisturizing and collagen-increasing effects of Pm-ME in HaCaT and HDF cells. (**a**,**b**,**e**,**f**) Expression levels of moisturizing-related genes (*HAS-2*, and *TGM-1*) in HaCaT cells (**a**) and *Col1A1* in HDF cells (**b**) treated with Pm-ME (0–100 µg/mL) for 24 h, or *Col1A1* in HDF cells exposed to UVB radiation (30 mJ/cm2) (**e**) or H_2_O_2_ (50 µg/mL) (**f**) in the presence or absence of Pm-ME (0–100 µg/mL) for 24 h were determined by RT-PCR. (**c**,**d**) *Col1A1*-promoter-derived luciferase activity from both HEK293 (**c**) and HDF (**d**) cells transfected with *Col1A1*-Luc in the presence or absence of Pm- ME (0–100 µg/mL) was determined by luminometer. ** *p* < 0.01 versus a control group (induced group).

Pm-ME enhanced the expression of moisturizing-related genes such as *HAS-2*, and *TGM-1* in HaCaT cells (Figure 3a). To determine if Pm-ME could enhance the expression of a collagen gene (*Col1A1*), we first treated HDF cells with Pm-ME (0–100 µg/mL) and then determined the expression level of *Col1A1*. As Figure 3b shows, an increased expression of the *Col1A1* gene under Pm-ME treatment conditions was dose-dependently observed. These results were further confirmed using a reporter gene assay with the Col1A1-Luc construct transfected in HEK293 cells and HDF cells. Expectedly, Pm-ME increased the promoter activity of the *Col1A1* gene in a dose-dependent manner in both cells (Figure 3c,d). In addition, Pm-ME was found to recover collagen gene level which decreased under both UV irradiation and H_2_O_2_ treatment conditions (Figure 3e,f).

### 2.4. Anti-Melanogenic Effect of Pm-ME

**Figure 4:**
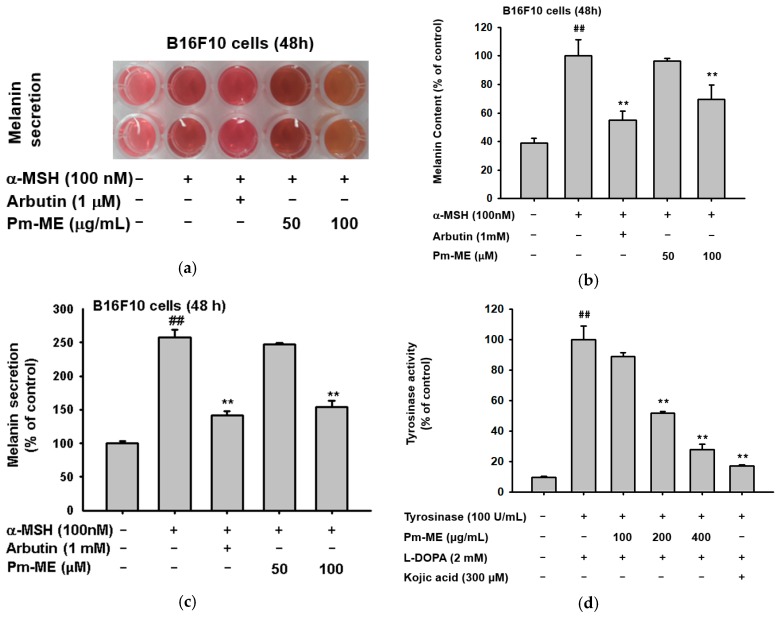
The effect of Pm-ME on melanogenesis in α-melanocyte stimulating hormone (MSH)-stimulated B16F10 cells. (**a**–**c**) B16F10 cells (1 × 105 cells/mL) were treated with α-MSH in the presence or absence of Pm-ME (0–100 µg/mL) or 1 mM arbutin for 48 h. Melanin content was determined by measuring the absorbance of the growth medium at 475 nm. For determining melanin secretion, the cell pellets were lysed, and the absorbance of B16F10 cell lysates was measured at 405 nm. (**d**) Effects of Pm-ME (0–400 µg/mL) and Kojic acid (KA) (300 µM) on tyrosinase’s activity were evaluated by tyrosinase assay with purified tyrosinase and L-DOPA. ## *p* < 0.01 versus a normal (untreated) group, ** *p* < 0.01 versus a control (induced) group.

Since our target concentration corresponded to 100 µg/mL, we determined whether Pm-ME was able to suppress secretion of melanin and its cellular contents. To determine this, B16F10 melanoma cells were stimulated by α-melanocyte stimulating hormone (α-MSH) in the presence or absence of Pm-ME or arbutin (positive control). Levels of melanin were then measured. Pm-ME reduced the secretion of melanin up to 75% at 100 µg/mL (Figure 4a,b). Pm-ME also decreased the melanin contents in MSH-treated B16F10 cells up to 30% at 100 µg/mL, whereas melanin content at 50 µg/mL was not significantly reduced, compared to the positive control (α-MSH) (Figure 4c). Finally, to determine the effect of Pm-ME on the activity of melanin-producing enzymes, we performed a tyrosinase enzyme assay. Interestingly, Pm-ME (0–400 µg/mL) significantly decreased tyrosinase activity at concentrations higher than 200 µg/mL, while Kojic acid (KA), a control drug, strongly reduced the activity of tyrosinase (Figure 4d).

### 2.5. Molecular Mechanisms of Pm-ME-Mediated Antiphotoaging and Moisturizing Effects

**Figure 5:**
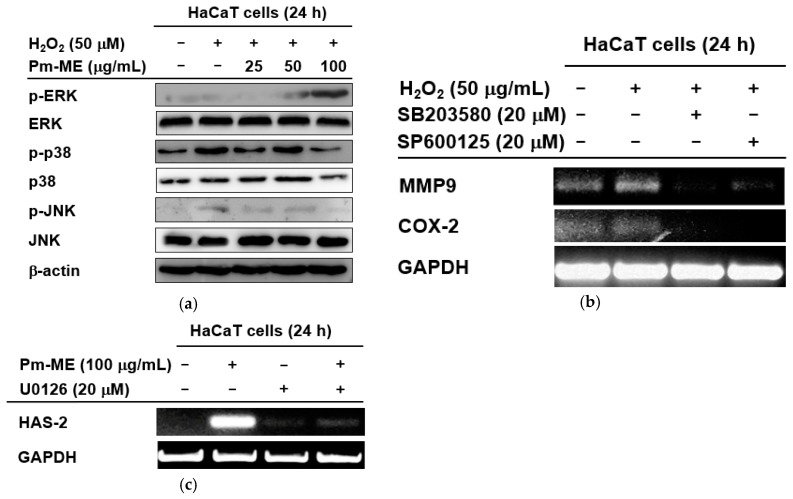
The effect of Pm-ME on the activation of MAPK in HaCaT cells. (**a**) HaCaT cells pre-treated for 30 min with Pm-ME (0–100 µg/mL) were treated with H_2_O_2_ (50 µM) for 24 h. Total and phospho-form levels of ERK, p38, and JNK were measured by Western blot. (**b**) HaCaT cells pre-treated with SB203580 (20 µM) or SP600125 (20 µM) for 30 min were treated with H_2_O_2_ (50 µM) for 24 h. The expression levels of *MMP-9* and *COX-2* were measured using semi-quantitative RT-PCR. (**c**) HaCaT cells pre-treated with U0126 (20 µM) for 30 min were treated with Pm-ME (100 µg/mL) for 24 h. The mRNA expression levels of *HAS-2* and *COL1A1* were determined by semi-quantitative RT-PCR.

To better understand which signaling molecules contributed to the anti-photoaging and moisturizing effects of Pm-ME, activation levels of MAPK were examined. MAPK-related enzymes are critical in regulating free radical-induced aging processes. H_2_O_2_-induced free radicals strongly increased the phosphorylation of p38 and JNK, but not ERK (Figure 5a). Conversely, Pm-ME increased levels of phospho-ERK, but decreased levels of phospho-p38 and phospho-JNK (Figure 5a). Importantly, the inhibition of JNK by SP600125, and that of p38 by SB203580, was linked to the suppression of *MMP-9* and *COX-2* (Figure 5b). Moreover, the Pm-ME-mediated expression of *HAS-2* was completely suppressed by U0126, an ERK inhibitor and recovered while treated with Pm-ME (200 µg/mL), suggesting a strong moisturizing activity (Figure 5c). 

## 3. Discussion

Because of their richness in nutrients and vitamins, different species from the Sapotaceae family have been used in traditional medicine [39]. However, the potential use of *P. mutisii* in the cosmetic and pharmaceutical industries has yet to be explored. For this reason, we studied the skin-protective activity of *P. mutisii* in keratinocytes and fibroblasts under skin-damaging conditions. We also evaluated its suitability for cosmetic preparations. Because toxicity testing to identify potential risks in humans is a necessary and critical step in both the drug and cosmetic industries [40], the cytotoxicity of *P. mutisii* was tested in HaCaT, B16F10, and HDF cells. *Pradosia mutisii* exhibited low toxicity untill 200 µg/mL and no toxicity at 100 µg/mL in HaCaT, B16F10 (Figure 1a,b), and HDF cells (Figure 3c). Using UHPLC/MS analysis (Figure 1c), Pm-ME was shown to have a high concentration of coumaric acid, a phenolic acid [41,42]. Polyphenols are secondary metabolites of plants and are generally involved in defenses against UV radiation or infection by pathogens. In the human body, polyphenols have been shown to protect against the development of cancer, cardiovascular diseases, diabetes, osteoporosis, and neurodegenerative diseases [1]. Coumaric acid has been shown to have antioxidant and anti-inflammatory properties [43].

Ultraviolet irradiation is one of the main causes of free radical (e.g., ROS) production in the skin. Free radicals can lead to skin aging and cancer if they accumulate over long periods of time [44]. It is known that free-radical-generated oxidative stress and damage can compromise cell survival, proliferation, differentiation, and metabolism [45,46]. Consistent with previous reports [47,48], we also found that UVB irradiation induced cellular damage in HaCaT cells. Damage included reduced proliferation, increased apoptosis, and molecular responses including the expression of genes related to inflammation, wrinkle formation, and aging (Figure 2 and Figure 3). Because UVB-induced radicals are major factors that damage cells, tissues, and organs, it was found that some endogenous compounds such as melatonin and its metabolite [49,50,51,52], and bilirubin [53] are involved in scavenging toxic radicals by exhibiting antioxidant, photo-protective, and anti-aging properties in our bodies. So far, these compounds are also developed as highly valuable biomaterials which can be applied to our body by pharmaceutical industries [54]. Nonetheless, vitamin C, coenzyme Q10, and α-tocopherol are becoming top global sales products for human health because of their significant anti-oxidative properties. Research laboratories at pharmaceutical and cosmetic companies continue to place a major emphasis on identifying and developing effective anti-oxidative drugs and natural compounds. In our study, we observed that Pm-ME had strong antioxidant activity when tested using the ABTS assay (Figure 2i). Similarly, Pm-ME reversed the free radical-induced suppression of cell adherence, the induction of nuclear damage, and the altered expression of genes such as *MMP-1*, *MMP-9*, and *COX-2* under UVB and H_2_O_2_ conditions in a dose-dependent manner (Figure 2a–f). Notably, Pm-ME restored levels of the *Sirt-1* gene (Figure 2g,h), which is involved in preventing apoptosis and increasing cell survival under oxidative stress conditions [26]. These results strongly implied that the aging of cells was linked to oxidative stress via the downregulation of *Sirt1* expression. The anti-oxidative properties of Pm-ME allowed for recovery of *Sirt1* expression. This may result in protection from skin damage and aging under natural UVB irradiation conditions in vivo. In addition, there is a possibility that Pm-ME is also able to protect UVC-induced cellular responses, since our UV irradiation conditions might be contaminated with UVC, although we used a filter system to clear out all wave lengths below 290 nm. The concept that anti-oxidative biomaterials can ameliorate cellular and molecular damage under oxidative stress conditions has driven the commercial production of various products such as BIOGF1K, ginsenoside Ro, EGCG, *Fraxinus chinensis* extract, and certain synthetic antioxidants. The anti-wrinkle and anti-photoaging effects of biomaterials in these and other products have been widely reported in the cosmetic industry [48,55,56]. 

Maintaining adequate skin hydration [31] and generating new collagen [57] are important processes for healthy skin. The expression of moisturizing (*HAS2*, *TGM-1*) (Figure 3a) and collagen (*Col1A1*) genes was remarkably increased by Pm-ME (Figure 3b–d), and helped to recover collagen levels after UVB exposure and H_2_O_2_ treatment (Figure 3e,f). Therefore, this suggests that Pm-ME can be an effective ingredient in the maintenance of healthy skin. The suppression of melanin by Pm-ME is also beneficial for its use in cosmetic preparations. Although melanin is valuable in protecting skin from UV irradiation, because many women prefer to lighten their skin, whitening by anti-melanogenic activity is a useful feature in cosmetic materials [29]. Pm-ME (100 µg/mL) displayed strong inhibitory activity during melanogenesis, as assessed by determination of melanin levels from secreted medium and the decrease shown in melanin secretion levels (Figure 4a–c). Moreover, Pm-ME (200 µg/mL) significantly suppressed tyrosinase enzyme activity (Figure 4d), implying that higher concentration is directly effective to suppress enzyme activity, while lower concentration is involved in regulation of melanin secretion. Therefore, because of its anti-oxidative, anti-photoaging, anti-wrinkle, moisturizer-stimulating, and anti-melanogenic activities, Pm-ME can be considered a good cosmeceutical candidate. To further explore this possibility, we plan to evaluate its clinical efficacy in future studies.

We performed studies to determine the molecular mechanisms by which Pm-ME exerted its anti-oxidative and moisturizing in keratinocytes. We focused on MAPK-related enzymes (ERK, JNK, and p38), because these enzymes play a critical role in the aging and melanogenesis of skin keratinocytes and melanocytes [58,59]. For example, the ERK pathway mediates cellular responses to transforming growth factor-β1 by increasing collagen [60] and HA synthesis pathways in keratinocytes [61]. JNK plays a key role in oxidative-stress-induced keratinocyte apoptosis [62]. The enzyme p38 is important in maintaining the homeostasis of human skin [63]. The JNK and p38 pathways both mediate cellular responses to cytokines and physical stress [14]. These pathways are activated by UVB- and ROS-induced stresses. These pathways can increase the production of proteolytic *MMP* and *COX-2* genes [15], which are related to collagen degradation and skin inflammation, respectively [64]. Notably, oxidative stress blocked the phosphorylation of ERK, while p38 and JNK were phosphorylated when treated with H_2_O_2_ (Figure 5a). In contrast, Pm-ME increased ERK phosphorylation and reduced the phosphorylation of p38 and JNK (Figure 5a), implying that these enzymes can differentially participate in Pm-ME-mediated pharmacological activities. To further understand the functional role of MAPK in the anti-photoaging, anti-melanogenic, and anti-inflammatory effects of Pm-ME in UVB-irradiated and H_2_O_2_-stimulated keratinocytes, specific inhibitors of MAPK were used. The results of Figure 5b strongly suggested that inhibition of p38 and JNK by Pm-ME resulted in anti-wrinkle and anti-inflammatory effects, because a p38 inhibitor (SB203580) and a JNK inhibitor (SP600125) blocked the expression of *MMP-9* and *COX-2*, respectively. Moreover, Pm-ME-triggered ERK phosphorylation induced the expression of genes related to moisturizing because Pm-ME treatment triggered the expression of HAS-2, whereas U0126 suppressed it (Figure 5c). These results suggested that MAPK-related enzymes contributed to PM-ME-mediated anti-aging, anti-wrinkle, and anti-inflammatory activities. UVB irradiation is known to increase other signaling cascades, such as those linked to NF-κB and STAT3 [65,66]. Whether Pm-ME can regulate these signaling pathways must still be investigated.

**Figure 6:**
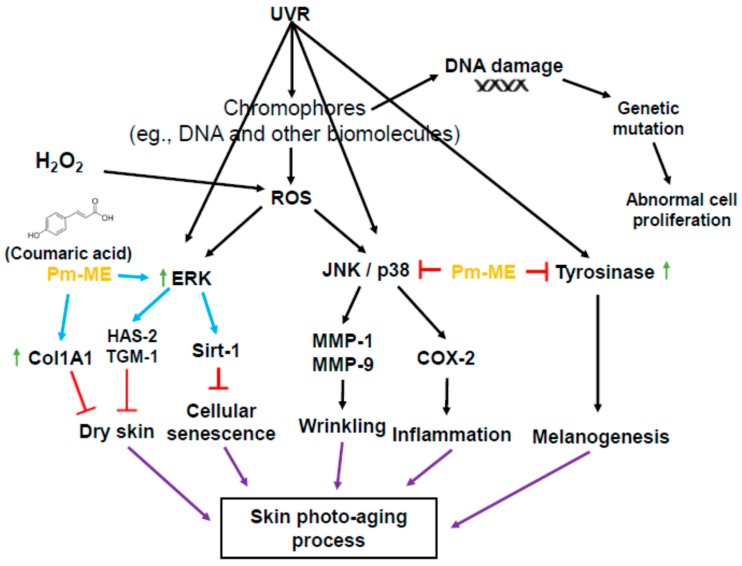
Schematic summary of the Pm-ME-mediated skin-protective pathway.

In conclusion, we found that coumaric acid-rich Pm-ME exhibited anti-oxidative, anti-wrinkle, moisturizer-stimulating, and anti-melanogenic activities in skin cells irradiated by UVB or treated with H_2_O_2_. Pm-ME increased the phosphorylation of ERK in a dose-dependent manner, but decreased the phosphorylation of p38 and JNK (Figure 6). Our results strongly suggested that Pm-ME may be an effective skin-protective biomaterial. Since Pm-ME exhibited very promising activities in both cancerous skin cells (HaCaT and B16F10 cells) and normal human keratinocytes during oxidative stress conditions, therefore, we propose the beneficial roles of Pm-ME’s use in cosmetic preparations. However, melanoma cells have abnormal features such as disrupted cell cycle control, aberrant gene expression patterns, and limited differentiation capacities [67], and additional work with primary melanocytes should be followed to exactly understand anti-melanogenesis activity of Pm-ME and its molecular mechanism. Moreover, plants from the same family (Sapotaceae) or genus (*Pradosia spp*.) will be tested for similar skin-protective activities.

## 4. Materials and Methods

### 4.1. Materials

The HaCaT, B16F10, and HEK293 cells were purchased from the American Type Culture Collection (Rockville, MD, USA). Neonatal, primary HDF cells (SKU: FC-0001) were obtained from Lifeline (Oceanside, CA, USA). The MTT, fetal bovine serum (FBS), phosphate-buffered saline (PBS), penicillin, and Dulbecco’s modified Eagle’s medium (DMEM) were purchased from Gibco (Grand Island, NY, USA). L-3,4-dihydroxyphenylalanine (L-DOPA), 5-hydroxy-2-hydroxymethyl-4H-pyranone (kojic acid), monophenol monooxygenase (mushroom tyrosinase), 4-hydroxyphenyl-β-D-glucopyranoside (arbutin), α-melanocyte stimulating hormone (α-MSH), polyethylenimine (PEI), 1-diphenyl-2 picryl-hydrazyl (DPPH), 2,2′-azino-bis (3-ethylbenzothiazoline-6-sulphonic acid) diammonium salt (ABTS), hydrogen peroxide (H_2_O_2_), retinol (RE), ascorbic acid (AA), and 6-diamidino-2-phenylindole (DAPI) were purchased from Sigma–Aldrich (St. Louis, MO, USA). TRIzol reagent was purchased from Thermo Fisher Scientific (Waltham, MA, USA). The primer sets for the polymerase chain reaction were synthetized by Macrogen (Seoul, Korea), and PCR premix was purchased from Bio-D Inc. (Seoul, Korea). Phospho- or total forms of p38, ERK, JNK, and β-actin were obtained from Cell Signaling Technology (Beverly, MA, USA).

### 4.2. Compound Analysis from Pm-ME by UHPLC, Coupled to Negative Electrospray Ionization High-Resolution Tandem Mass Spectrometry (UPLC/HRMS)

A 95% methanol extract of *P. mutisii* (Pm-EE) was purchased from Korea Plant Extract Bank (Daejeon, Korea, http://extract.kribb.re.kr/, E-mail: plantext@kribb.re.kr). Compound analysis was performed by UPLC/HRMS (Orbitrap) analyses using Shimadzu Ultra Performance LCMS 8050 system (Shimadzu, Kyoto, Japan) with a triple quadrupole mass spectrometer equipped with electrospray ionization (ESI) source operating in negative mode. Lab Solutions software version 5.2 (Shimadzu) was used as reported previously [68,69]. The sample solutions were injected into a reversed phase column (BEH C8, 1.7 µm, 2.1 mm × 150 mm, Waters, Milford, MA, USA) with appropriate pre-columns. The column was maintained at 40 °C. The mobile phase consisted of the mixture of aqueous solutions of 10 mM formic acid (solvent A) and acetonitrile (solvent B) at a flow rate of 0.25 mL/min. The linear gradient and isocratic flows of the mobile phase were 5% B for 0.8 min, 5–10% B for next 0.4 min, isocratic 10% B for 0.70 min, 10–15% B for next 0.5 min, isocratic 15% B for 1.30 min, 15–21% B for 1.30 min, isocratic 21% B for 1.20 min, 21–27% B for next 0.50 min, then 27–50% B for 3.30 min, 50–100% B for 2.00 min, isocratic 100% B for 1.00 min, and 100–5% B over 5 min. At the end of the program, the column was equilibrated under the initial conditions for 2.70 min. The pressure ranged from 45 to 50 MPa during the chromatographic run. The effluent was introduced into an electrospray source (interface temperature 300 °C, heat block temperature 400 °C, and capillary voltage 3.0 kV). Argon was used as the collision gas and nitrogen as the nebulizing gas. The interface between the liquid chromatography and the mass spectrometry detector was conducted using ESI. After precursor ion full scan in the negative ion mode (i.e., [M-H]^−^), the product ions were determined using tandem mass spectrometry. To achieve a high specificity in addition to the high sensitivity, we used an analysis in the multiple reaction monitoring mode.

### 4.3. Cell Culture

HaCaT cells (a human keratinocyte cell line), B16F10 cells (a murine melanocyte cell line), and HDF cells (a human fibroblast cell line) were cultured in DMEM supplemented with 10% FBS and 1% penicillin–streptomycin, while HEK293 cells (a human embryonic kidney cell line) were incubated in DMEM supplemented with 5% FBS and 1% penicillin–streptomycin. All cells were kept at 37 °C in a 5% humidified incubator.

### 4.4. Cell Viability Assay

The HaCaT cells were seeded at a density of 4 × 10^4^ cells per well in a 96-well plate for 24 h, and then treated with Pm-ME for 24 h. The B16F10 cells were seeded at a density of 1 × 10^4^ cells per well in a 96-well plate and treated under the same conditions. The HDF cells were seeded at a density of 1 × 10^5^ cells per mL in a 96-well plate for 24 h. Cell viability for the previously mentioned cell lines was measured using the MTT assay, in which cells were first incubated with 10 µL/well of MTT solution for 3 h and then treated with 100 µL of MTT stopping solution (10% sodium dodecyl sulfate with 10% HCl). After 8 h, the absorbance of the solubilized formazan was measured at 570 nm using an optical density reader (BioTek, Winooski, VT, USA).

### 4.5. Free Radical Scavenging Activity

The radical scavenging activity of Pm-ME was determined using ABTS assay. The ABTS assay was performed as reported previously [70]. Briefly, 7.4 mM ABTS and 2.4 mM potassium persulfate solutions were mixed at a 1:1 ratio and incubated at room temperature overnight to generate ABTS radicalization. Then different concentrations of Pm-ME (0–200 µg/mL) or AA (100 µg/mL) were mixed with the ABTS solution and transferred to a 96-well plate, followed by an incubation period of 30 min at 37 °C. The absorbance was measured at 730 nm. The ABTS scavenging effect was calculated as follows:

ABTS scavenging effect (%) = [(A0−A1)/A100] × 100(1)

where A0 is the absorbance of ABTS, and A1 is the absorbance of samples.

### 4.6. DAPI Staining

The HaCaT cells were seeded at a density of 4 × 10^5^ cells/mL in a 12-well plate containing previously sterilized, round glass cover slips. After 24 h, cells were treated with Pm-ME for 30 min, washed with PBS, and treated with H_2_O_2_ (50 µM) for 24 h. Cells were washed twice with PBS and fixed with 1 mL of 3.7% paraformaldehyde in PBS for 10 min. Cells were washed with PBS two more times, stained with DAPI reagent (1 µL/mL) for 30 min, and then washed with PBS two more times. The cover slip was then mounted on a rectangular glass slide using mounting solution and left to dry at room temperature for 24 h [71]. Samples were examined using a Nikon Eclipse Ti fluorescence microscope (Nikon, Tokyo, Japan). 

### 4.7. UVB Irradiation and the Morphological Change Assay

The HaCaT cells were seeded at a density of 4 × 10^5^ cells/mL in a 6-well plate. Cells were treated with Pm-ME for 30 min, washed with PBS, and then subjected to 30 mJ/cm^2^ of UVB radiation (absorbance peak at 312 nm) using a UVB lamp (Bio-link BLX-312, Vilber Lourmat, Collegien, France) fitted with a Kodak Kodacel K6808^®^ filter that eliminates all wavelengths below 290 nm, as reported previously [72]. After UVB treatment, cells were treated for 24 h with Pm-ME according to previous papers [36]. Morphological changes were assessed using an inverted phase-contrast microscope (Olympus, Tokyo, Japan) attached to a video camera with NIH imaging software (Bethesda, Maryland, USA).

### 4.8. Semi-Quantitative RT-PCR Analysis

The HaCaT cells were seeded at a density of 4 × 10^5^ cells/mL in a 12-well plate. For UVB analysis, cell treatments were carried out as previously described in the previous section. For H_2_O_2_ treatment, cells were also seeded at a density of 4 × 10^5^ cells/mL in a 12-well plate, treated with Pm-ME for 30 min, washed with PBS, and then treated with H_2_O_2_ (50 µM) for 24 h. To determine the Pm-ME moisturizing effect on HaCaT cells, they were first treated with the compound (0–100 µg/mL) and then subjected to mRNA extraction. For determining the role of MAPK in skin ageing and moisturizing, HaCaT cells were pretreated for 30 min with 20 µM of SB203580 (p38 inhibitor), SP600125 (a JNK inhibitor), followed by incubation with H_2_O_2_ (50 µM) for 24 h, and mRNA was extracted and the levels of *MMP-9* and *COX-2* were quantified. For determining ERK’s role, HaCaT cells were treated with 20 µM of ERK inhibitor (U0126) alone or with Pm-ME (100 µg/mL) for 24 h, after which *HAS-2* levels were measured. For determining the effect of Pm-ME on *Col1A1* expression, HDF cells were seeded at a density of 1 × 10^5^ cells per mL in a 6-well plate for 24 h, treated with Pm-ME (0–100 µg/mL) for 24 h, and then subjected to mRNA extraction. For determining if Pm-ME could recover gene expression of collagen decreased after UVR and ROS exposure, HDF cells were seeded at a density of 1 × 10^5^ cells per mL in a 6-well plate for 24 h, treated with Pm-ME (0–100 µg/mL) for 30 min, subjected to H_2_O_2_ (50 µM) or UVB radiation (30 mJ/cm^2^), and further cultivated with Pm-ME (0–100 µg/mL) for 24 h. Total mRNA was extracted using TRIzol reagent according to the manufacturer’s instructions. Semiquantitative RT-PCR assay was carried out using MuLV reverse transcriptase, as previously described [37]. The total RNA (1 µg) was incubated with oligo-dT15 at 70 °C for 5 min and mixed with a 5× first-strand buffer, 10 mM of dNTPs and 0.1 M dithiothreitol, then further incubated at 37 °C for 5 min, and for 60 min following the addition of MuLV reverse transcriptase (2 U). The reactions were finished at 70 °C for 10 min and the total RNA was removed by adding RNase H. The PCR reaction was conducted with the incubation mixture (2 µL cDNA, 4 M 5′ and 3′ primers, 10× buffer (10 mM Tris–HCl, pH 8.3, 50 mM KCl, 0.1% Triton X-100), 250 µM of dNTPs, 25 mM of MgCl2 and 1 unit of Taq polymerase (Promega, Madison, WI, USA)) under the following incubation conditions: a 45 s denaturation time at 94 °C, annealing time of 45 s at 55–60 °C, extension time of 60 s at 72 °C and final extension of 7 min at 72 °C after 25–30 cycles. The primers (Bioneer, Seoul, Korea) used in this experiment are listed in Table 1.

### 4.9. Plasmid Transfection and Luciferase Reporter Gene Assay

For the luciferase reporter gene assay, HEK293 and HDF cells were first seeded at a density of 1 × 10^5^ cells/well in 24-well plates. Both cell lines were then transfected with pCMV0Red Firefly Luc plasmids containing 1 kb of Col1A1 promoter region and β-galactosidase (as a transfection control) genes (0.8 µg/mL). Transfection was achieved using the PEI method for 24 h. This was followed by treatment with the compound (0–100 µg/mL) for a further 24 h. Retinol (10 µg/mL), a *Col1A1* gene upregulating compound [60], was used as a positive control. Luciferase activity was measured according to the Luciferase Assay System (Promega), as previously reported [37]. Cell lysates were centrifuged at maximum speed for 10 min in an Eppendorf microcentrifuge. Then, 50 µL of the supernatant fraction was incubated with 50 µL of luciferase substrate, and the relative luciferase activity was determined with a Luminoskan Ascent (Thermo Labsystems Oy, Helsinki, Finland). Luciferase activity was normalized to β-galactosidase activity, and measured at 405 nm, by enzymatic reaction with X-gal and lysate for 5 min at 37 °C.

### 4.10. Melanogenesis and Melanin Secretion Assays

The B16F10 cells were treated with α-MSH (100 nM) and either Pm-ME (0–100 µg/mL) or arbutin (1 mM) for 48 h. To determine the melanin secretion from cells, the absorbance of the cell culture medium was measured at 475 nm using a Spectramax 250 microplate reader (Molecular Devices, San Jose, CA, USA). Cells were washed with cold PBS and harvested. For measurement of melanin content, cells were lysed with 20 mL cell lysis buffer (50 mM Tris-HCl pH 7.5, 20 mM NaF, 25 mM β-glycerolphosphate pH 7.5, 120 mM NaCl, and 2% NP-40 in distilled water) and centrifuged at 12,000 rpm for 10 min. The supernatants were removed and the pellet was dissolved in 100 µL 1 M NaOH containing 10% DMSO at 60 °C for 30 min. The absorbance of each fraction was measured at 405 nm using a Spectramax 250 microplate reader (Molecular Devices, San Jose, CA, USA) [36].

### 4.11. Tyrosinase Assay

For determining the tyrosinase enzyme activity, 50 mL of L-DOPA, 50 mL of Pm-ME (0–400 µg/mL) or 300 µM of Kojic acid were incubated for 15 min with mushroom tyrosinase (100 U/mL) at room temperature. Absorbance of each sample was measured at 475 nm using a Spectramax 250 microplate reader (Molecular Devices, San Jose, CA, USA).

### 4.12. Western Blot Analysis

The HaCaT cells were pretreated with Pm-ME (0–100 µg/mL) for 30 min and then treated with H_2_O_2_ (50 µM) for 24 h. Cell lysates were prepared as previously described by Park et al. [73]. Lysates were subjected to sodium dodecyl sulfate–polyacrylamide gel electrophoresis followed by transfer to polyvinylidene fluoride membranes. Using specific antibodies, total and phosphorylated forms of target proteins were detected and visualized by chemiluminescence reagents.

### 4.13. Statistical Analysis

All data are presented as the mean ± standard deviation of at least three independent experiments. A Mann–Whitney test was used to compare statistical differences between experimental and control groups. A *p*-value < 0.05 was considered statistically significant. All statistical analyses were conducted using SPSS program (SPSS, Chicago, IL, USA).

## Figures and Tables

**Table 1:** Sequences of primers (human) used in semi-quantitative RT-PCR.

Name	Primer	Sequence (5′ to 3′)
*MMP-1*	Forward	TCTGACGTTGATCCCAGAGAGCAG
	Reverse	CAGGGTGACACCAGTGACTGCAC
*MMP-9*	Forward	GCCACTTGTCGGCGATAAGG
	Reverse	CACTGTCCACCCCTCAGAGC
*Col-1A1*	Forward	CAGGTACCATGACCGAGACG
	Reverse	AGCACCATCATTTCCACGAG
*COX2*	Forward	CAAAAGCTGGGAAGCCTTCT
	Reverse	CCATCCTTCAAAAGGCGCAG
*HAS-2*	Forward	CCACCCAGTACAGCGTCAAC
	Reverse	CATGGTGCTTCTGTCGCTCT
*Sirt-1*	Forward	CAGTGTCATGGTTCCTTTGC
	Reverse	CACCGAGGAACTACCTGAT
*TGM-1*	Forward	AGGGAAGATCCAAGAGCCCA
	Reverse	ACTCTGGATCCCCTACGCTT
*GAPDH*	Forward	CACTCACGGCAAATTCAACGGCAC
	Reverse	GACTCCACGACATACTCAGCAC

## Data Availability

The data used to support the findings of this study are available from the corresponding author upon request.

## Abbreviations

Pm-ME	*P. mutisii methanol* extract
MMPs	matrix metalloproteinases
α-MSH	α-melanocyte-stimulating hormone
ROS	reactive oxygen species
KA	kojic acid
L-DOPA	L-3,4-dihydroxyphenylalanine
UV	ultraviolet light
DAPI	6-diamidino-2-phenylindole
H_2_O_2_	hydrogen peroxide
MAPK	mitogen-activated protein kinases
ERK	extracellular signal-regulated kinase
JNK	c-Jun-N-terminal kinase
MTT	3-(4,5-dimethylthiazol-2-yl)-2,5-diphenyltetrazolium bromide
ABTS	2,2’-Azino-bis (3-ethylbenzothiazoline-6-sulphonic acid) diammonium salt
AA	ascorbic acid
RT-PCR	reverse transcription-polymerase chain reaction
